# Semi-Synthetic Analogues of Cryptolepine as a Potential Source of Sustainable Drugs for the Treatment of Malaria, Human African Trypanosomiasis, and Cancer

**DOI:** 10.3389/fphar.2022.875647

**Published:** 2022-04-26

**Authors:** Yabalu Z. Abacha, Arnold Donkor Forkuo, Stephen Y. Gbedema, Nimisha Mittal, Sabine Ottilie, Frances Rocamora, Elizabeth A. Winzeler, Donelly A. van Schalkwyk, John M. Kelly, Martin C. Taylor, Janette Reader, Lyn-Marie Birkholtz, David R. Lisgarten, Jeremy K. Cockcroft, John N. Lisgarten, Rex A. Palmer, Rosemary C. Talbert, Steven D. Shnyder, Colin W. Wright

**Affiliations:** ^1^ School of Pharmacy and Medical Sciences, University of Bradford, Bradford, United Kingdom; ^2^ Department of Pharmacognosy, Faculty of Pharmacy, University of Maiduguri, Maiduguri, Nigeria; ^3^ Department of Pharmacology, Faculty of Pharmacy and Pharmaceutical Sciences, College of Health Sciences, Kwame Nkrumah University of Science and Technology (KNUST), Kumasi, Ghana; ^4^ Department of Pharmaceutics, Faculty of Pharmacy and Pharmaceutical Sciences, College of Health Sciences, KNUST, Kumasi, Ghana; ^5^ Malaria Drug Accelerator (MalDA) Consortium, School of Medicine, University of California, San Diego, La Jolla, CA, United States; ^6^ Department of Infection Biology, Faculty of Infectious and Tropical Diseases, London School of Hygiene and Tropical Medicine, London, United Kingdom; ^7^ Department of Biochemistry, Genetics and Microbiology, Faculty of Natural and Agricultural Sciences, Institute for Sustainable Malaria Control, University of Pretoria, Hatfield, South Africa; ^8^ Biomolecular Research Group, School of Psychology and Life Sciences, Canterbury Christ Church University, Canterbury, United Kingdom; ^9^ Department of Chemistry, Christopher Ingold Laboratories, University College London, London, United Kingdom; ^10^ School of Science, University of Greenwich, Chatham, United Kingdom; ^11^ Department of Crystallography, Biochemical Sciences, Birkbeck College, University of London, London, United Kingdom; ^12^ School of Pharmacy and Medical Sciences, Institute of Cancer Therapeutics, University of Bradford, Bradford, United Kingdom

**Keywords:** sustainable pharmaceuticals, halogenation of cryptolepine, *Plasmodium falciparum*, *Plasmodium knowlesi*, *Trypanosoma brucei*, ovarian cancer

## Abstract

The prospect of eradicating malaria continues to be challenging in the face of increasing parasite resistance to antimalarial drugs so that novel antimalarials active against asexual, sexual, and liver-stage malaria parasites are urgently needed. In addition, new antimalarials need to be affordable and available to those most in need and, bearing in mind climate change, should ideally be sustainable. The West African climbing shrub *Cryptolepis sanguinolenta* is used traditionally for the treatment of malaria; its principal alkaloid, cryptolepine (**1**), has been shown to have antimalarial properties, and the synthetic analogue 2,7-dibromocryptolepine (**2**) is of interest as a lead toward new antimalarial agents. Cryptolepine (**1**) was isolated using a two-step Soxhlet extraction of *C. sanguinolenta* roots, followed by crystallization (yield 0.8% calculated as a base with respect to the dried roots). Semi-synthetic 7-bromo- (**3**), 7, 9-dibromo- (**4**), 7-iodo- (**5**), and 7, 9-dibromocryptolepine (**6**) were obtained in excellent yields by reaction of **1** with *N*-bromo- or *N*-iodosuccinimide in trifluoroacetic acid as a solvent. All compounds were active against *Plasmodia in vitro*, but **6** showed the most selective profile with respect to Hep G2 cells: *P. falciparum* (chloroquine-resistant strain K1), IC_50_ = 0.25 µM, SI = 113; late stage, gametocytes, IC_50_ = 2.2 µM, SI = 13; liver stage, *P. berghei* sporozoites IC_50_ = 6.13 µM, SI = 4.6. Compounds **3**–**6** were also active against the emerging zoonotic species *P. knowlesi* with **5** being the most potent (IC_50_ = 0.11 µM). In addition, **3**–**6** potently inhibited *T. brucei in vitro* at nM concentrations and good selectivity with **6** again being the most selective (IC_50_ = 59 nM, SI = 478). These compounds were also cytotoxic to wild-type ovarian cancer cells as well as adriamycin-resistant and, except for **5**, cisplatin-resistant ovarian cancer cells. In an acute oral toxicity test in mice, **3**–**6** did not exhibit toxic effects at doses of up to 100 mg/kg/dose × 3 consecutive days. This study demonstrates that *C. sanguinolenta* may be utilized as a sustainable source of novel compounds that may lead to the development of novel agents for the treatment of malaria, African trypanosomiasis, and cancer.

## 1 Introduction

Although global malaria deaths declined steadily from 2000 to 2019, estimated deaths from malaria in 2020 increased by 12% compared to 2019 (627,000 in 2020 compared with 558,000 in 2019), of which an estimated 47,000 deaths resulted from disruption due to the Covid-19 pandemic (World Malaria Report, 2021). In the same period, malaria cases increased from an estimated 227 million in 2019 to 241 million in 2020. Recent evidence of the independent emergence of partial artemisinin resistance in the African WHO region is of great concern as is the development of resistance to partner artemisinin combination therapy (ACT) drugs in the Greater Mekong WHO sub-region, especially if this were to happen in Africa (World Malaria Report, 2021). Although the RTS,S malaria vaccine has now been approved for the prevention of *P. falciparum* malaria in children in regions where moderate-high transmission occurs, the current situation underlines the need for novel antimalarial drugs in order to achieve the Global technical strategy aim of reducing malaria incidence and deaths by 90% over the period 2016–2030 (WHO, 2016). Furthermore, the long-term goal of malaria eradication will require antimalarials with enhanced product profiles that are effective not only against asexual blood-stage parasites but also against liver schizonts, hypnozoites, and gametocytes for chemoprotection, relapse prevention, and transmission blocking, respectively (Burrows et al., 2017). In addition, new antimalarials should be affordable and available to those who need them most, and it may also be argued that in the context of climate change/global warming, it is important that their production should not be environmentally damaging.

In addition to malaria, other neglected tropical diseases continue to impose a major health and economic burden on the developing world. For example, African trypanosomiasis, which is caused by parasites of the *Trypanosoma brucei* species complex, is endemic in 36 sub-Saharan African countries. Although public health and control measures have greatly reduced human infections in recent times, the disease has considerable epidemic potential and is a major constraint on development through its impact on domestic livestock (WHO, 2021). Traditionally, the drugs used to treat *T. brucei* infections have been characterized by limited efficacy and/or significant toxicity (Dickie et al., 2020). The recent introduction of the nitroheterocyclic compound fexinidazole as an oral treatment for both stage 1 and stage 2 infections has been a major step forward (Pelfrene et al., 2019). However, there remains a need for alternative therapies and there is a huge scope for improved veterinary drugs.

Historically, the natural products exemplified by quinine and artemisinin have made a huge contribution to the chemotherapy of malaria and may have the potential to be more environmentally “friendly” than synthetic compounds. The roots of the West African climbing shrub *Cryptolepis sanguinolenta* (Lindl.) Schltr. (Apocynaceae) are used in the traditional medicines for the treatment of malaria and other infectious and non-infectious diseases (Shnyder and Wright, 2021), and although this species has traditionally been collected from the wild, importantly, efforts to cultivate the plant are now being made (Amissah et al., 2016). The indoloquinoline alkaloid cryptolepine (**1**) is the major alkaloid present in the roots of this species and is of interest as a lead to new antimalarial drugs. In the previous work, **1** was shown to have modest antiplasmodial activity against chloroquine-sensitive (HB3) and resistant (K1) strains of *P. falciparum in vitro* as well as oral activity against *P. berghei* in mice (80.5% suppression of parasitemia compared to the untreated infected controls at 50 mg/kg/day) (Wright et al., 1996). However, **1** has been shown to inhibit DNA synthesis and topoisomerase II (Bonjean et al., 1998) and to intercalate into DNA with a preference for non-alternating G-C sites (Bonjean et al., 1998; Lisgarten et al., 2002), and there is evidence that **1** may carry a genotoxic risk (Ansah et al., 2005; Gopalan et al., 2011). It was also shown that the antiplasmodial mode of action of **1** involves a chloroquine-like mode of action (inhibition of hemozoin formation) that is independent of interactions with DNA (Wright et al., 2001), suggesting the possibility that it may be possible to prepare analogues of **1** that do not intercalate into DNA but retain antiplasmodial properties. A number of halogenated analogues of **1** were synthesized, and 2, 7-dibromocryptolepine (**2**) was found to have antiplasmodial activities 10-fold higher than **1** and also suppressed parasitemia in mice by 90% when given I.P. at 25 mg/kg without apparent toxicity to the mice (Onyeibor et al., 2005). Thermodenaturation studies showed that in contrast to **1,** analogue **2** did not intercalate into DNA (ΔT_m_ values of 4 and 9°C for **2** and **1**, respectively), with values below 5°C, indicating non-specific binding to DNA, suggesting that DNA intercalation is not involved in the antiplasmodial action of **2**. However, like **1,** compound **2** was shown to inhibit hemozoin formation, but experiments have indicated that the increased potency of **2** is neither due to more potent inhibition of hemozoin formation nor due to increased accumulation of this basic compound into the acidic parasite food vacuole compared to **1**, suggesting that a second, currently unknown mechanism of action may be operating (Onyeibor et al., 2005). Taken together, these data suggest that **2** may be a lead to novel antimalarial agents, but as chemical synthesis is not environmentally friendly, an alternative approach utilizing *C. sanguinolenta* roots is desirable. Here, we report the isolation of **1** using a “green” extraction procedure, followed by the semi-synthesis of halogenated analogues **3**–**6** and their evaluation for antiplasmodial activity against chloroquine-sensitive (Dd2) and resistant (K1) strains of *P. falciparum* and *P. knowlesi* (A1-H.1) asexual blood stages. In addition, **2**, **4**, **5**, and **6** were assessed for the activity against *P. berghei* sporozoites/Hep G2 cells, and **1**, **2,** and **6** were tested against late-stage *P. falciparum* gametocytes; **2** was also evaluated for resilience against resistance development. As **2** has previously been shown to have promising *in vitro* and *in vivo* activities against *T. brucei* (Oluwafemi et al., 2009), **3**–**6** were also tested against *T. brucei in vitro*. Compounds **1** and **3**–**6** were also evaluated for cytotoxic activity against wild-type, adriamycin-resistant, and cisplatin-resistant ovarian cancer cells, and **3**–**6** were assessed for acute oral toxicity in mice. Overall, this study aimed to explore the feasibility of utilizing the roots of *C. sanguinolenta* as a sustainable source of leads to novel antimalarial, antitrypanosomal, and anticancer drugs.

## 2 Materials and Methods

### 2.1 Materials

The sun-dried, cut roots of *Cryptolepis sanguinolenta* used in this study were obtained from the Centre for Plant Medicine Research (formally called the Centre for Scientific Research into Plant Medicine), Mampong-Akwapim, Ghana, in August 2012 and were identified at the Plant Development Centre of the institution. Its authenticity was confirmed by Dr. G. H. Sam of the KNUST Herbal Medicine Department and subsequently compared to a voucher specimen KNUST/HM1/2008/L056 at the KNUST Herbal Medicine Department herbarium in Kumasi.

Solvents (reagent grade) were obtained from Fisher Scientific, United Kingdom. Chemicals (reagent grade), except where stated below, were purchased from Merck (Sigma Aldrich), United Kingdom.

### 2.2 Extraction and Isolation of Cryptolepine (1)

Dried, powdered roots of *C. sanguinolenta* (50 g) were extracted with ethyl acetate in a Soxhlet extractor until the solvent returning to the flask became colorless. The plant material was removed from the Soxhlet, allowed to dry, moistened with ammonium hydroxide solution, 35% (25 ml), and returned to the Soxhlet extractor, and extraction was repeated with ethanol until the returning solvent was no longer reddish purple in color. The experiments were also carried out using chloroform as both the first and second solvent. The extract was filtered (VWR qualitative filter paper 413) to remove particles of the plant material, concentrated under reduced pressure using a rotary evaporator (45°C), and dried *in vacuo* at 30°C. The residue was acidified with citric acid solution (10% ^w^/_v_ in ethanol) and then refluxed with the addition of sufficient ethanol to just dissolve the residue. The solution was filtered hot, concentrated using a rotary evaporator to approximately 50 ml, and then placed in a freezer at −18°C for 24–48 h. The mother liquor was carefully removed; the crystallized cryptolepine citrate was washed with a small quantity of cold ethanol and then dried *in vacuo*. A second batch of crystals was obtained in the same way following the concentration of the remaining mother liquor to a small volume.

### 2.3 Preparation of Synthetic Cryptolepine Analogue 2

2, 7-Dibromocryptolepine (**2**) was synthesized using 5-bromoindoxyldiacetate and 5-bromoisatin as starting materials, as previously described (Wright et al., 2001).

#### 2.3.1 Semi-Synthesis of Cryptolepine Analogues 3–6

7-Bromocryptolepine (**3**): Cryptolepine (**1**), (20 mg base, 0.086 mM) was dissolved in trifluoroacetic acid (TFA) (3 ml) at room temperature, and 1 equivalent of *N-*bromosuccinimide (NBS) (15.3 mg, 0.086 mM, dissolved in TFA, 2 ml) was added dropwise with stirring over 10–15 min (Figure 2). The product formation was monitored by thin-layer chromatography using analytical silica gel G_F254_ TLC plates (Merck, mobile phase chloroform: methanol: ammonium hydroxide 35%, 8:2:0.1; plates were examined in daylight and under UV light at 254 and 365 nM). The reaction mixture was diluted with water (20 ml), basified with ammonium hydroxide solution 35%, and partitioned with ethyl acetate (3 × 10 ml). The organic layer was washed with water twice, dried over anhydrous sodium sulfate, filtered, evaporated under reduced pressure, and dried *in vacuo*.

7, 9-Dibromocryptolepine (**4**): As for **3** above except that up to 4 equivalents of NBS were required for complete reaction. 7-iodocryptolepine (**5**) and 7, 9-diiodocryptolepine (**6)** were prepared as above except that *N*-iodosuccinimide (NIS) was used in place of NBS. Hydrochloride salts of **3**–**6** were prepared by addition of HCl 35%, (5% ^v^/_v_) in ethanol to solutions of **3**–**6** in ethanol, and citrate salts were similarly prepared by the addition of citric acid, (10% ^w^/_v_) in ethanol. Solutions of **3**–**6** were concentrated using a rotary evaporator and crystallized at −18°C for 24–48 h.

### 2.4 Characterization of Compounds

^1^H-NMR spectra at 400 MHz and ^13^C NMR spectra at 100.6 MHz and correlation experiments were acquired using a Bruker DP 400 MHz NMR spectrometer. Low-resolution mass spectrometry (LRMS) was carried out on a Quattro Ultima Triple Quad Mass Spectrometer (Micromass) in electrospray positive and negative modes. Accurate mass measurements (HRMS) were obtained using a Thermo Orbitrap LTQ XL.

### 2.5 X-Ray Crystallography of 6 as a Hydrochloride Salt

Needle-shaped crystals were obtained after 3 weeks by slow evaporation of a solution of **6** in methanol: ethanol (50:50) at room temperature. A suitable crystal was selected and mounted on a SuperNova, Dual, Cu at zero, Atlas diffractometer. The crystal was flash-frozen and kept at 150°K during data collection. Data collection was carried out using monochromatic CuK radiation on a SuperNova Cu X-ray Source). Data were processed during data collection with the program CrysAlisPro, Agilent Technologies Version 1.171.36.28.

### 2.6 *In Vitro* Culture and Antiplasmodial Activity of Blood-Stage Malaria Parasites

*P. falciparum* (3D7 clone) and *P. knowlesi* (A1-H.1 clone) were maintained in culture in RPMI 1640 supplemented with 25 mM HEPES, 25 mM NaHCO_3_, 10 mM D-glucose, 2 mM L-glutamine, 50 mg/L hypoxanthine, 25 mg/L gentamicin sulfate, 5 g/L Albumax II, and 10% (v/v) equine serum (Thermo Fisher Scientific, 26-050-088). The *P. falciparum* (3D7) and *P. knowlesi* (A1-H.1) parasites were grown in human A^+^ red blood cells (National Health Blood and Transplant, United Kingdom). The parasites were incubated in sealed flasks at 37°C under a culture gas mixture of 96% N_2_, 3% CO_2_, and 1% O_2_. (van Schalkwyk et al., 2019). The compounds were initially dissolved in DMSO prior to diluting in RPMI. All experiments were initiated using unsynchronized parasites with both the parasitemia and hematocrit set to 1%. Drug susceptibility assays were set up in 96-well, flat-bottom microplates in a final volume of 200 μL, as described previously (van Schalkwyk et al., 2017). The controls were included for the background fluorescence (0% viability; parasites exposed to a supralethal 10 μM chloroquine concentration) and 100% growth (parasites in drug-free wells). The plates were incubated for one complete life cycle (27 h for *P*. *knowlesi* or 48 h for *P*. *falciparum in vitro*) at 37°C in a modular incubation chamber (Billups-Rothenburg Inc.) under the culture gas. Upon termination of the assay, the plates were stored at −20°C overnight. The SYBR Green I fluorescent method was used to measure parasite survival (Bennett et al., 2004; Smilkstein et al., 2004). After the microplates were thawed, 100 μL from each well was transferred into a duplicate plate. To this was added 100 μL of SYBR green solution [SYBR Green I (Thermo Fisher Scientific, S7563), diluted 1:5000 in a lysis buffer made of 20 mM Tris, 5 mM EDTA, 0.008% (w/v) saponin, 0.08% (v/v) Triton X-100, pH 7.5]. The plates were stored in the dark for 1 h before fluorescence was read at a 490 nm excitation wavelength and a 520 nm emission wavelength on a Spectramax M3 microplate reader (Molecular Devices) (van Schalkwyk et al., 2017). The background fluorescence (wells with parasites at 0% viability) was subtracted from all drug assay wells, and the parasite proliferation was determined as a percentage of the fluorescence in the drug-free control wells.

*P. falciparum* (K1 clone) was maintained in human erythrocytes prepared from the donated blood samples (blood groups unknown, Ethical Tissue, University of Bradford), suspended in the RPMI 1640 medium as previously described (Wright et al., 2001), except that serum was replaced with Albumax II, [5% (^w^/_v_)]. The compounds were dissolved in 100% DMSO and further diluted with the RPMI 1640 medium (the final DMSO concentration did not exceed 0.5%, which did not affect parasite growth). Twofold serial dilutions were made in 96-well microtiter plates in duplicate, and infected erythrocytes were added to give a final volume of 100 µL with 2.5% hematocrit and 1% parasitemia. Chloroquine diphosphate was used as a positive control, and uninfected and infected erythrocytes without compounds were included in each test. The plates were placed into a modular incubator; gassed with 93% nitrogen, 3% oxygen, and 4% carbon dioxide; and incubated at 37°C for 48 h. The parasite growth was assessed by measuring lactate dehydrogenase activity as described by Makler et al. (1993). The reagent used contained the following in each mL: acetylpyridine adenine dinucleotide (APAD), 0.74 mg; lithium lactate, 19.2 mg; diaphorase, 0.1 mg; Triton X-100, 2 μL; nitroblue tetrazolium, 1 mg; and phenazine ethosulphate, 0.5 mg. Fifty microliter of this reagent was added to each well and mixed; plates were incubated for 15 min at 37°C. The optical densities were read at 550 nM using an Azure 96-well microplate reader (Azure Biosystems Inc.), and % inhibition of growth was calculated by comparison with the control values. IC_50_ values were determined using linear regression analysis (Microsoft Excel). A minimum of three separate determinations was carried out for each compound.

### 2.7 Cultivation and *In Vitro* Evolution of Drug Resistance in *P. falciparum*

The Dd2 strain of *P. falciparum* was used for *in vitro* drug selection. Continuous parasite cultivation was performed under standard conditions as previously described by Trager and Jensen, (1976). The parasites were grown in human O-positive (O^+^) whole blood obtained from the Blood Bank of The Scripps Research Institute (TSRI) (La Jolla, CA, United States). Leukocyte-free erythrocytes were washed and then stored at 50% hematocrit in RPMI 1640. The evaluation of parasitemia and the parasite morphology was performed using a microscopic evaluation of thin blood smears that were first fixed with methanol (Merck) and then stained with Giemsa (Sigma). Generation of *in vitro*-evolved resistant parasites was attempted two times on a parental Dd2 strain of *P. falciparum*. The first round of selection involved continuous exposure of 10^8^ parasites to a starting concentration of 30 nM (3 × IC_50_) and eventually increased up to 4 × IC_50_ over the course of 90 days. The second round of selection was performed by exposing 10^8^ Dd2 parasites to a stepwise increasing concentration of the drug starting from 1 × IC_50_ up to 4 × IC_50_ over the course of 90 days. The flasks were maintained at 1% hematocrit.

### 2.8 *P. berghei* Liver-Stage Assay

The liver-stage activities of crytolepine analogues **2** and **4**–**6** were tested as previously described (Antonova-Koch, et al., 2018). Briefly, 3000 Hep G2-A16-CD81 cells/well were seeded in 1536-well plates (Greiner Bio). 50 nL of the test and control compound diluted in DMSO was added and incubated for 24 h. Thereafter, *P. berghei* sporozoites (*P. berghei* ANKA GFP-Luc-SMcon) freshly obtained by dissecting salivary glands of infected *A. stephensi* mosquitoes were added to each well at a density of 1 × 10^3^ per well. The plates were centrifuged for 5 min at 330 × g and incubated at 37°C. Forty-eight hours post infection, 2 μL of luciferin reagent (Promega BrightGlo) was added to each well, and the luciferase activity was detected using a Perkin Elmer Envision plate reader. IC_50_ values were determined in CDD vault (https://www.collaborativedrug.com/) normalized to maximum and minimum inhibition levels for the positive (atovaquone 0.25 μM) and negative (DMSO) control wells. To counter-screen for cellular toxicity, Hep G2-A16-CD81 cells were seeded as above for the sporozoite liver-stage assay but in the absence of sporozoites. Toxicity measurements were performed by adding Promega CellTiterGlo^®^ (2 μL), followed by luminescence measurements. Curve fitting was done as described above using puromycin (25 μM) as a positive control and DMSO as negative control wells.

### 2.9 *In Vitro* Activity of 1, 2, and 6 Against Late Gametocyte Stages of *P. falciparum*

Gametocytes were produced as per Reader et al. (2015; 2021) from *P. falciparum* NF54-Mal8p1.16-GFP-Luc (Adjalley et al., 2011), and each batch was validated for viability and functionality before use. The luciferase reporter assay was established to enable accurate, reliable, and quantifiable investigations of the stage-specific action of gametocytocidal compounds. Drug assays were set up on day 10 (representing late gametocytes, >90% stage IV/V gametocytes) using a 2–3% gametocytemia, 1.5% hematocrit culture. The drug pressure was for 48 h under stationary, hypoxic conditions (90% N_2_, 5% O_2_, and 5% CO_2_) at 37°C. Luciferase activity was determined in 30 μL parasite lysates by adding 30 μL luciferin substrate (Promega Luciferase Assay System) at room temperature and detection of resultant bioluminescence at an integration constant of 10 s with the GloMax^®^-Multi + Detection System with Instinct^®^ Software. Methylene blue (5 μM) and an internal control (MMV390048, 5 μM) were routinely included as controls.

### 2.10 *In Vitro* Activity of 3–6 Against *T. brucei*

Bloodstream form *T. brucei* (strain 427, derivative 221) was maintained in modified Iscove’s medium, as described previously (Taylor et al., 2013). Seven-point potency curves were performed in 96-well microliter plates (200 μL volumes), and the compound concentrations that inhibited growth by 50% (IC_50_) and 90% (IC_90_) were determined. The parasites were diluted to 1 × 10^4^ ml^−1^, the compound was added at a range of concentrations, and the plates were incubated at 37°C in 5% CO_2_ for 48 h. Resazurin was then added to each well, and the plates were incubated for a further 16 h. Fluorescence intensities were determined using a BMG FLUOstar Omega plate reader (excitation 545 nm, emission 590 nm), and data were analyzed using Graph Pad Prism 9.0 software. The values are expressed as IC_50_ ± SD and are the average of three independent replicates.

### 2.11 Chemosensitivity in Mammalian Cancer Cells

The chemosensitivity of compounds **3**–**6** was assessed in the wild-type immortalized human ovarian adenocarcinoma cell line A2780 and its daughter cell lines, which are resistant to the standard cancer chemotherapeutics doxorubicin (A2780/ADR) and cisplatin (A2780/CIS, all cell lines from ECACC, Salisbury, United Kingdom). A modified MTT assay was used (Shnyder et al., 2008). Cells (2 × 10^4^/ml) were inoculated into each well of a 96-well plate and incubated for 24 h at 37°C in a humidified atmosphere containing 5% CO_2_. Compounds were then added at a range of concentrations to each well, and the plates were then incubated for a further 96 h. After 96 h, the culture medium was removed and 200 μL of 0.5 mg ml^–1^ MTT solution (Sigma) in the complete medium was added to each well. Following a further 4 h incubation, the solution was removed from each well and 150 μL of DMSO was added to solubilize the formazan crystals resulting from MTT conversion. The absorbance values for the resulting solutions were read at 540 nm on an Azure 96-well microplate reader (Azure Biosynstems Inc.), and the cell survival was calculated as the absorbance of treated cells divided by the control. The results were expressed in terms of IC_50_ values (i.e., the concentration of the compound required to kill 50% of cells), and all experiments were performed in triplicate.

### 2.12 Evaluation of Acute Toxicity in Mice

Female NIH Swiss mice aged 6–12 weeks were used (Envigo, Blackthorn, United Kingdom). The mice were kept in cages housed in isolation cabinets in an air-conditioned room with regular alternating cycles of light and darkness. They received Teklad 2018 diet (Envigo, Blackthorn, United Kingdom) and water *ad libitum*. All animal procedures were carried out under a project license issued by the UK Home Office, and UK National Cancer Research Institute Guidelines for the Welfare of Animals were followed throughout. Compounds **2** (as hydrochloride) and **4**–**6** (as citrate salts) were dissolved in 10% DMSO/water on the day of treatment as described above and administered orally to groups of two mice in a volume of 0.1 ml per 10 g body weight on 3 consecutive days (days 0–2) at three dose levels (20, 50, and 100 mg/kg/day). A group of two untreated control animals was included at each dose level. Following treatment, the body weight was measured on a regular basis, and behavior and general appearance were monitored visually to assess for deleterious effects (e.g., dehydration, impaired mobility, hunched posture, a low body temperature, ulceration, and significant bodyweight loss), with any effects during the study recorded. If the bodyweight loss was >15% over a 72 h period or if animal behavior and appearance were significantly altered, then mice were immediately sacrificed by cervical dislocation. If no deleterious effects were seen after at least 18 days of study, then the animals were sacrificed, and the dose was considered non-toxic.

### 2.13 Statistical Analysis

Data are presented as the mean ± standard error (SE) of at least three independent experiments.

## 3 Results and Discussion

### 3.1 Development of a “Green” Method for the Extraction and Isolation of Cryptolepine

In the previous work, the extraction and isolation of **1** from *C. sanguinolenta* involved moistening the dried powdered roots with ammonium hydroxide, extraction of the base with chloroform, followed by chromatography over aluminum oxide, and eluting with chloroform: methanol 95:5 (Wright et al., 1996). The method described here avoids the use of toxic solvents and eliminates the need for adsorbents, thus reducing waste. In the first stage of the process, Soxhlet extraction with ethyl acetate removed all relatively non-polar compounds from the plant material but did not elute the alkaloids as they are present as salts in the plant material. Following basification of the plant material with ammonium hydroxide solution, the alkaloids, predominantly **1**, were extracted by repeating the Soxhlet extraction using ethanol. The latter was chosen for the basic extraction as ethyl acetate was unsuitable (presumably due to the release of acetic acid on refluxing), while **1** appeared to be degraded when acetone/ammonium hydroxide was used. Crystallization of **1** from ethanol as the citrate salt yielded 0.8% cryptolepine (as the base) with respect to the dried roots. The yield of **1** was less than that obtained when chloroform was used for both the first and second extractions (up to 1.0%) and comparable to previous reports in which **1** was isolated from Ghanaian samples of *C. sanguinolenta* using methods that utilized chlorinated solvents (Grellier et al., 1996, yield 0.76%; Tackie et al., 1993, yield 0.48%). Although *C. sanguinolenta* has been reported to contain up to 15 indoloquinoline alkaloids, apart from **1**, these are present in small amounts (Shnyder and Wright, 2021). The ^1^H-NMR spectrum (Supplementary Figures S1A, S1B) indicates that the crystallized product is of good purity with only traces of minor alkaloids and the polar material.

### 3.2 Semi-Synthesis and Characterization of Cryptolepine Analogues

**FIGURE 1:**
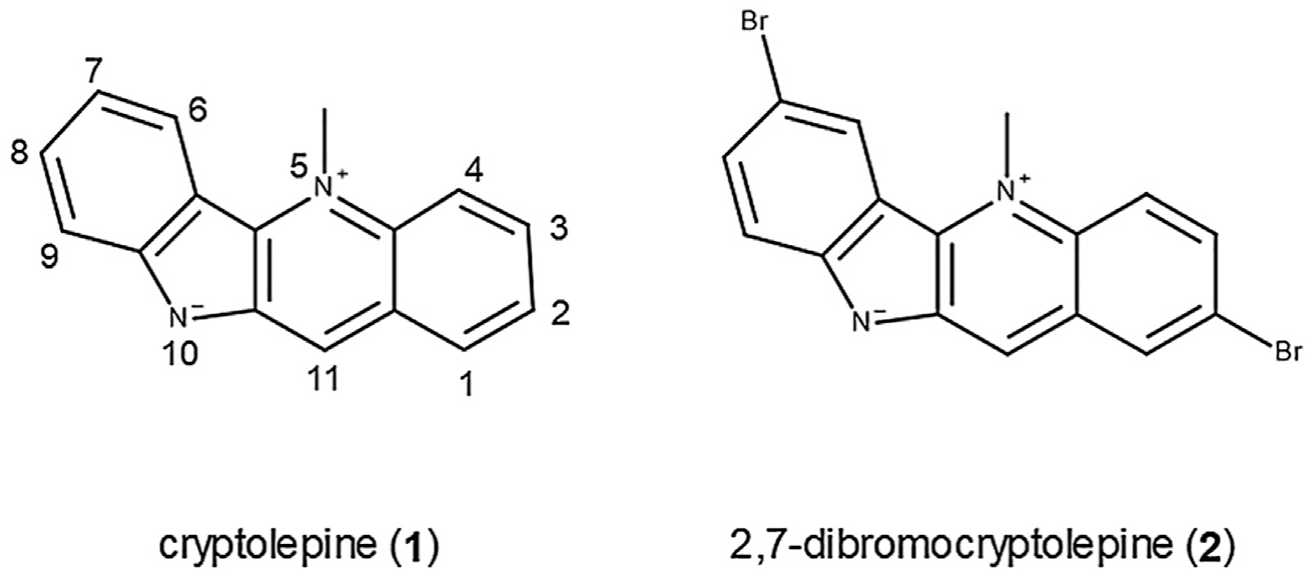
Structures of cryptolepine (**1**) and 2, 7-dibromocrytolepine (**2**).

The structures of cryptolepine (**1**) with the numbering scheme and the synthetic analogue 2,7-dibromocryptolepine (**2**) are shown in Figure 1.

**FIGURE 2:**
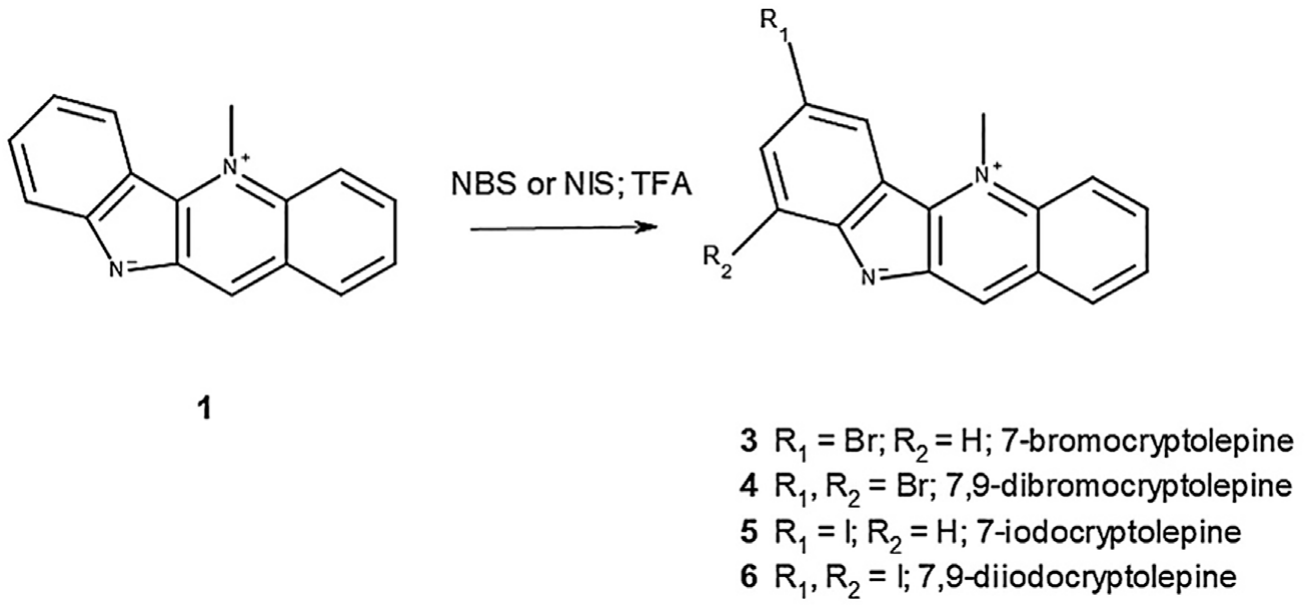
Semi-synthesis of brominated and iodinated derivatives of **1**.

The methodology for the semi-synthesis of **3**–**6** was adapted from the previous work by Castanet et al. (2002) and Bergström et al. (2017), in which it was shown that the direct region-selective iodination of aromatic compounds could be achieved with NIS in the presence of catalytic amounts of TFA or by using TFA as a solvent. In this study, it was found that attempts to iodinate **1** with catalytic quantities of TFA were unsuccessful, but the reaction with NIS took place rapidly at room temperature when the reactants were dissolved in TFA to give **5 (yield 74.5%)** and **6 (yield 85.6%)** depending on the equivalents of NIS used with respect to **1**. Similarly, bromination of **1** to give **3 (yield 86.7%)** and **4 (yield 89.6%)** was achieved with excellent yields (Figure 2).

**FIGURE 3:**
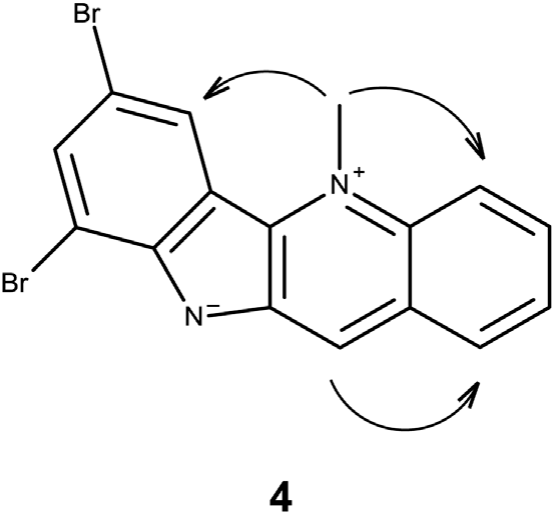
NOE correlations in **4**.

Spectroscopic data for **3** were found to be identical to 7-bromocryptolepine (Wright et al., 2001). Analogues **4**–**6** were shown to be the 7,9-dibromo-, 7-iodo-, and 7,9-diodo- derivatives of **1**, respectively, by means of ^1^H-NMR, ^13^C-NMR, and correlation NMR spectroscopy (Supplementary Figures S2–S4 and Supplementary Tables S1–S3). The molecular formulas for **4**–**6** were confirmed by accurate mass measurements (Supplementary Table S4). The substitution pattern was confirmed using nuclear Overhauser effect (NOE) spectroscopy (Figure 3). Using **4** as an example, an NOE correlation from the *N*-5 methyl group signal to proton 6 indicates that the substitution pattern must be 7, 9 rather than 6, 8.

**FIGURE 4:**
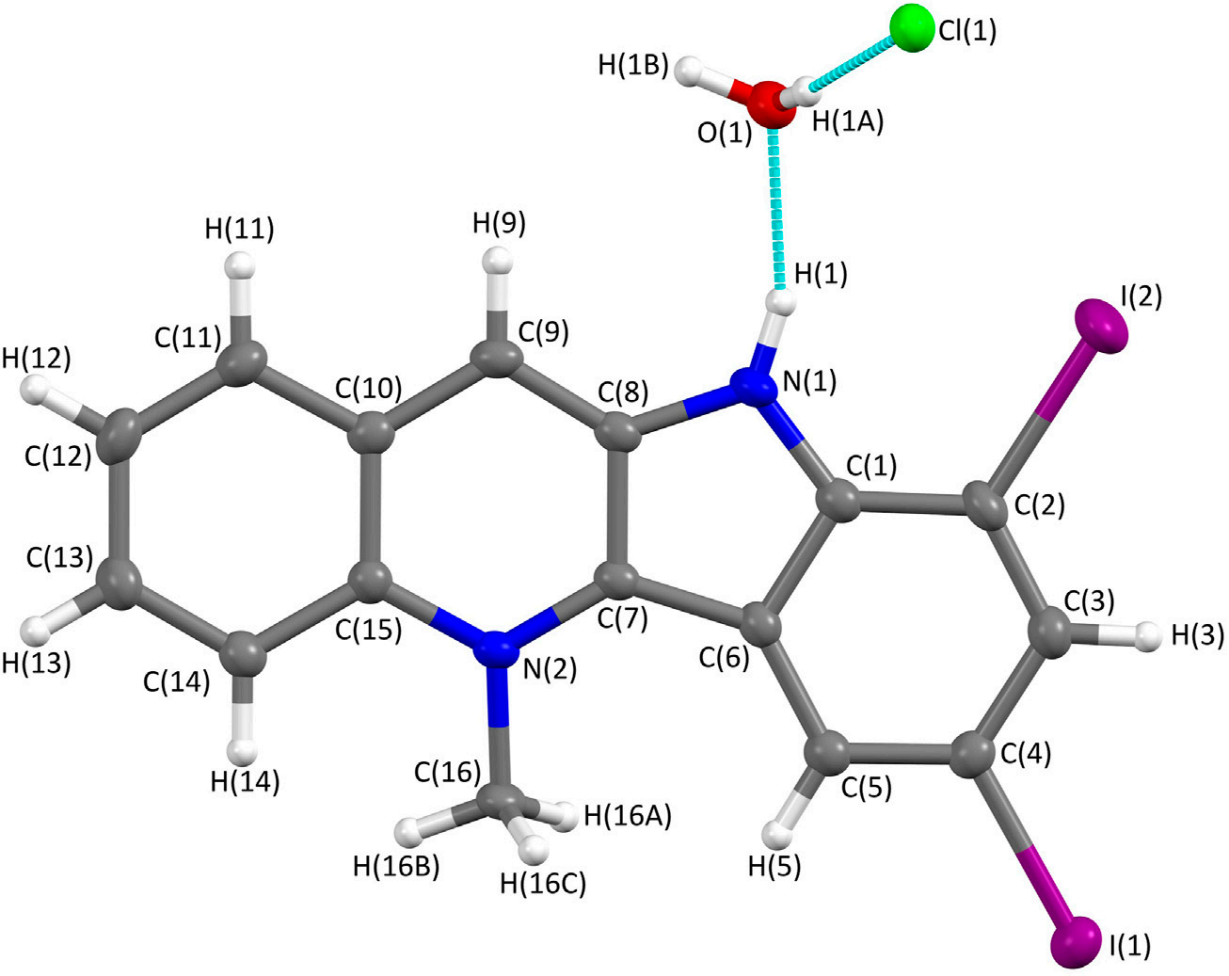
Molecular structure of 7,9-diiodocryptolepine (**6**) determined from X-ray single-crystal diffraction using Mo Kα radiation. The structure was solved within the Olex2 system using SHELXT and refined using SHELX with an *R*-factor of 0.027, showing a high level of agreement between the refined structure and the observed data. The crystal structure confirms 7, 9-diiodo-substitution of the indole ring; the protonation of nitrogen N (1) and the presence of a chloride anion indicate the hydrochloride salt; one molecule of water is associated with each molecule of **6**.

The structure of **6** was also unequivocally confirmed by means of X-ray crystallography (Figure 4, Supplementary Figure S5).

The reaction of **1** with NBS or NIS provides a more convenient route to 7- or 7, 9-brominated or iodinated semi-synthetic derivatives compared with total synthesis, although it is recognized that, as recently shown by Chen et al. (2021), greater compound diversity may be more easily achieved using total synthesis.

### 3.3 Activity Against Asexual Blood-Stage Parasites

The activities of cryptolepine (**1**) and the synthetic 2,7-dibromo- derivative (**2**) against *P. falciparum* chloroquine- and pyrimethamine-resistant strain K1 were found to be very similar to previously published values (IC_50_ = 0.44 and 0.049 µM, respectively, Table 1), (Onyeibor et al., 2005). The semi-synthetic analogue **4** was of similar activity to **1**, while **3**, **5**, and **6** were 1.8-3-fold more potent than **1** (Table 1). Although none of the compounds were as active as **2**, the selectivity of **6** with respect to Hep G2 cells was slightly higher than that of **2** (SI = 113 and 101, respectively, Table 1). All the compounds showed similar antiplasmodial activities against chloroquine-sensitive *P. falciparum* strain 3D7 as well as against the zoonotic parasite *P. knowlesi*, with both parasites being assayed under the same conditions. *P. knowlesi* was the predominant species in Malaysia in 2020 with 2,607 cases reported, while no cases of *P. falciparum* or *P. vivax* were observed (World Malaria Report, 2021). This species has been shown to cause severe and fatal disease (Barber et al., 2021), so it is important that this parasite is taken into account with respect to malaria drug development as species differences in drug susceptibility have been reported for other new antimalarial candidates (van Schalkwyk et al., 2019).

**TABLE 1:** *In vitro* activities of **1**–**6** against blood- and liver-stage malaria parasites.

Compound	Activity against *P. falciparum* K1, IC_50_, µM (SI)^a^	Activity against *P. falciparum* 3D7, IC_50_ µM	Activity against *P. knowlesi* A1-H.1, IC_50_, µM ± SD	Activity against late-stage gametocytes IC_50_, µM ± SE (SI)^a^	Activity against the *P. berghei* liver stage, IC_50_, µM ± SE (SI)^a^	Activity against Hep G cells; IC_50_ µM ± SE
1	0.44 ± 0.22	0.46 ± 0.04	1.04 ± 0.023	2.40 ± 0.30	NT	NT
2	0.049 ± 0.02 (101)^b^	0.03 ± 0.006	0.03 ± 0.0045	2.0 ± 0.17 (2.5)	3.72 ± 1.36 (1.32)	4.94 ± 1.81
3	0.15 ± 0.03	NT	NT	NT	NT	NT
4	0.44 ± 0.09 (15.4)	0.76 ± 0.06	1.12 ± 0.450	NT	5.82 ± 0.26 (1.17)	6.79 ± 0.92
5	0.16 ± 0.03 (41.9)	0.092 ± 0.02	0.104 ± 0.04	NT	14.6 ± 0.42 (0.46)	6.70 ± 0.50
6	0.25 ± 0.04 (113)	0.66 ± 0.06	1.04 ± 0.22	2.20 ± 0.02 (12.8)	6.13 ± 0.62 (4.6)	28.2 ± 4.10
Chloroquine diphosphate	0.35 ± 0.14	0.012 ± 0.003	0.02 ± 0.003	—	—	—
Methylene blue	—	—	—	96% inhibition at 5 µM	—	—
MMV 390048	—	—	—	96.2% inhibition at 5 µM	—	—
Atovaquone	—	—	—	—	0.000383	—

a Selectivity index with respect to Hep G cells.

b Data from Onyeibor et al., 2005. Each datum represents the mean ± SE of at least three independent experiments.

### 3.4 Gametocytocidal Assay

The inhibitory concentrations (full dose response) of compounds **1**, **2**, and **6** against *P. falciparum* (NF54-Mal8p1.16-GFP-Luc) late-stage gametocytes were determined using the luciferase reporter assay platform using the NF54-Mal8p1.16-GFP-Luc reporter line and are shown in Table 1. Dose–response curves and assay quality parameters are shown in Supplementary Figure S6.

For all three compounds, IC_50_ curves indicated moderate activity, with the IC_50_ of cryptolepine obtained from the luciferase assay platform used here, 2.4 ± 0.3 μM, correlating well with previous data where the IC_50_ of this compound was found to be 1.97 μM (Forkuo et al., 2017). Analogues **2** and **6** were found to be similarly active (IC_50_ = 2.0 and 2.2 µM, respectively), indicating that they retain the activity of **1**, but especially in **6**, this does not parallel the increase in activity observed against asexual blood-stage parasites. However, when compared with its cytotoxicity to Hep G2 cells, **6** exhibits better selective toxicity against gametocytes than **2** (SI = 13 and 2.5, respectively, Table 1). To further profile these compounds, we make the recommendation to test the compounds against the early stages of the gametocyte to investigate any stage specificity. Furthermore, these compounds can be interrogated for gametocidal activity by exploring their activity against the male and female gametes.

### 3.5 Activity Against Liver-Stage Malaria Parasites

The activities of compounds **2** and **4**–**6** against *P. berghei* sporozoites and the host Hep G cells are shown in Table 1. Dose–response curves of test and control compounds are shown in Supplementary Figure S7. Compounds **2** and **4** were the most active against sporozoites but were similarly toxic to Hep G cells, while **5** was ∼2-fold more toxic to Hep G cells; in contrast, **6** showed the best selectivity against sporozoites (SI = 4.6), and as shown above, **6** is also the most selective analogue against late gametocyte stages.

### 3.6 *In Vitro* Evolution of Drug Resistance in *P. falciparum*

Generation of *in vitro-*evolved resistant parasites of **2** was attempted twice on a parental Dd2 strain of *P. falciparum*. Although the parasites tolerated concentrations of up to 4 × IC_50_ under selection conditions, no resistant parasites were obtained from both attempts, suggesting that **2** is resilient to resistance development by *P. falciparum*. As discussed in the introduction, evidence suggests that **2** has a second, at present unknown antiplasmodial mode of action in addition to that of inhibition of hemozoin formation, which may explain, at least in part, its resilience to resistance development. Further studies using a hypermutator *Plasmodium* line would be worthwhile to further characterize the resilience observed in this study.

### 3.7 Antitrypanosomal Activity

In this study, 2,7-dibromocryptolepine (**2**) was found to have potent *in vitro* activity against *T. brucei* bloodstream forms with good selectivity (Table 2, IC_50_ = 16.7 nM, SI = 309) when compared with cytotoxicity against Hep G2 cells. These data are consistent with the previous work (Oluwafemi et al., 2009), in which **2** was shown to have potent, selective activity against *T. brucei* (strain 427) *in vitro* as well as oral activity against *T. brucei* infection in rats, while **1** was 100-fold less potent than **2** against *T. brucei in vitro*. In this study, the semi-synthetic analogues **4**–**6** exhibited potent *in vitro* antitrypanosomal activities with modest selectivity compared with their cytotoxicity against Hep G2 cells (Table 2), while **3** was 3- to 6-fold less active. In contrast, **4**–**6** did not show selective toxicity to trypanosomes when compared with their cytotoxic activities against ovarian cancer A2780 WT cells but did not exhibit oral toxicity to mice (see below).

**TABLE 2:** *In vitro* activities of 1–6 and positive controls against *T. brucei* bloodstream forms and ovarian cancer cell lines.

Compounds	Activity against *T. brucei* IC_50_, nM±SE (SI)^a^ ^,^ ^b^	Activity against *T. brucei* IC_90_, nM±SE^a^	Activity against A2780/WT cells, IC_50_, µM±SE	Activity against A2780/ADR cells, IC_50_, µM±SE	Activity against A2780/cis cells, IC_50_, µM±SE
1	306^c^	NT	0.44 ± 0.2	1.27 ± 0.6	0.67 ± 0.39
2	16.7 ± 0.7 (309)	39.7 ± 0.7	NT	NT	NT
3	199 ± 15	256 ± 6	0.07 ± 0.01	0.17 ± 0.04	0.23 ± 0.02
4	28.3 ± 0.5 (240)	40.3 ± 6.1	0.20 ± 0.58	0.31 ± 0.06	0.58 ± 0.1
5	81.3 ± 2.5 (82.4)	113 ± 11	0.06 ± 0.01	0.18 ± 0.01	0.31 ± 0.19
6	59 ± 13 (478)	114 ± 11	0.13 ± 0.07	0.18 ± 0.02	0.28 ± 0.05
Cisplatin	—	—	3.47 ± 2.2	NT	24.67 ± 2.75
Doxorubicin	—	—	0.07 ± 0.06	3.03 ± 0.35	NT

a Tested as hydrochloride salts except for 6, tested as citrate.

b Selectivity index compared with cytotoxicity to Hep G cells (Table 1).

c Data from Oluwafemi et al. 2009. Each datum represents the mean ± SE of at least three independent experiments.

### 3.8 Cytotoxicity Against Ovarian Cancer Cell Lines

Compounds **3**–**6** all demonstrated improved potency against the A2780 wild-type ovarian adenocarcinoma cell line when compared to the parent compound **1**, with compound **3** demonstrating the best chemosensitivity of the new analogues (Table 2). When considering drug resistance, there was only a slight decrease in sensitivity seen for compounds **3**–**6** in the A2780/ADR doxorubicin-resistant cell line when compared to the A2780/WT cell line (∼2–3-fold increase in IC_50_ compared to ∼40-fold for doxorubicin), suggesting that these analogues are not affected by doxorubicin-resistance mechanisms. For cisplatin, there was a similar decrease in sensitivity in the A2780/CIS cisplatin-resistant cell line when compared to the A2780/WT cell line for all the analogues apart from compound **5** (∼2–5-fold increase in IC_50_ compared to ∼7-fold for cisplatin), which would suggest that compounds **3**, **4**, and **6** are not affected by cisplatin-resistance mechanisms, but compound **5** may be impacted.

### 3.9 Acute Oral *In Vivo* Toxicity of Analogues 2 and 4–6

Semi-synthetic analogues **3**–**6** and synthetic analogue **2** were evaluated for oral toxicity in mice at doses of 20, 50, and 100 mg/kg administered for 3 consecutive days. No toxicity was seen for any of the analogues up to the highest dose of 100 mg/kg/dose (Supplementary Figure S8 **)**. A previously reported study with the parent compound **1** demonstrated that it could be administered safely orally to mice at 50 mg/kg/dose with an antimalarial effect (Wright et al., 1996), and therefore, the lack of toxicity at twice this dose suggests that there is a broad window for success for *in vivo* activity, assuming that the compounds are found to have sturdy pharmacokinetic properties.

## 4 Conclusion

An efficient method for the extraction and isolation of **1** from *C. sanguinolenta* roots based on a two-step Soxhlet extraction process, followed by crystallization of **1**, as the citrate salt has been developed without the need for toxic chlorinated solvents. Halogenated derivatives **3**–**6** were semi-synthesized from **1** in a one-step reaction by reaction with NBS or NIS in TFA as a solvent and evaluated for their antiplasmodial profiles. Analogues **3**–**6** were active against both chloroquine-sensitive and chloroquine-resistant *P. falciparum* asexual blood-stage parasites but were less active than the synthetic lead compound **2**. Cryptolepine (**1**) and **3**–**6** were also active against the emerging species *P. knowlesi*. Compound **6** exhibited similar activities to **1** and **2** against late-stage gametocytes, and **1**, **4**, and **6** showed moderate activity against *P. berghei* sporozoites. However, **6** was the most selective compound (with respect to Hep G2 cells) against asexual, liver, and gametocyte *Plasmodium* stages and was found to be superior to **2.** In addition, **4**–**6** exhibited potent (nM) activity against *T. brucei* with good selectivity with respect to Hep G2 cells. Against ovarian cancer cell lines, **1** and **3**–**6** were toxic against sensitive and drug-resistant cell lines, although **5** may be relatively less active against the cisplatin-resistant line. Interestingly, **2** and **4**–**6** were not toxic to mice at doses of up to 100 mg/kg/dose.

Taken together, these data show that it is feasible to isolate **1** from *C. sanguinolenta* roots using traditional, ecofriendly methods and to prepare semi-synthetic halogenated analogues as potential leads to novel drugs for the treatment of malaria, trypanosomiasis, and ovarian cancer. Further work aimed at improving the selectivity and optimizing the pharmacokinetic profiles of **3**–**6** would be worthwhile.

## Data Availability

The datasets presented in this study can be found in online repositories. The names of the repository/repositories and accession number(s) can be found below: X-ray data for compound **6**. The corresponding CIF file with hkl and intensity data have been deposited at the Cambridge Crystallographic Data Center at https://www.ccdc.cam.ac.uk with deposition codes 2155899 (Cu) and 2155900 (Mo).
