# Supplementary material for: Semi-Synthetic Analogues of Cryptolepine as a Potential Source of Sustainable Drugs for the Treatment of Malaria, Human African Trypanosomiasis, and Cancer
Source: Front Pharmacol. 2022 Apr 26;13:875647. doi: 10.3389/fphar.2022.875647 (PMC9119314; doi:10.3389/fphar.2022.875647)

Figure 1A. ¹H-NMR (400 MHz) of Cryptolepine (1) as Citrate in d_6-DMSO. NMR-signals of 1 are located above 5 ppm; signals due to the methylene groups in citrate are observed overlapping the DMSO signal at 2.5 ppm; the signal at 3.33 ppm is due to water in the solvent; other small peaks indicate traces of polar impurities.

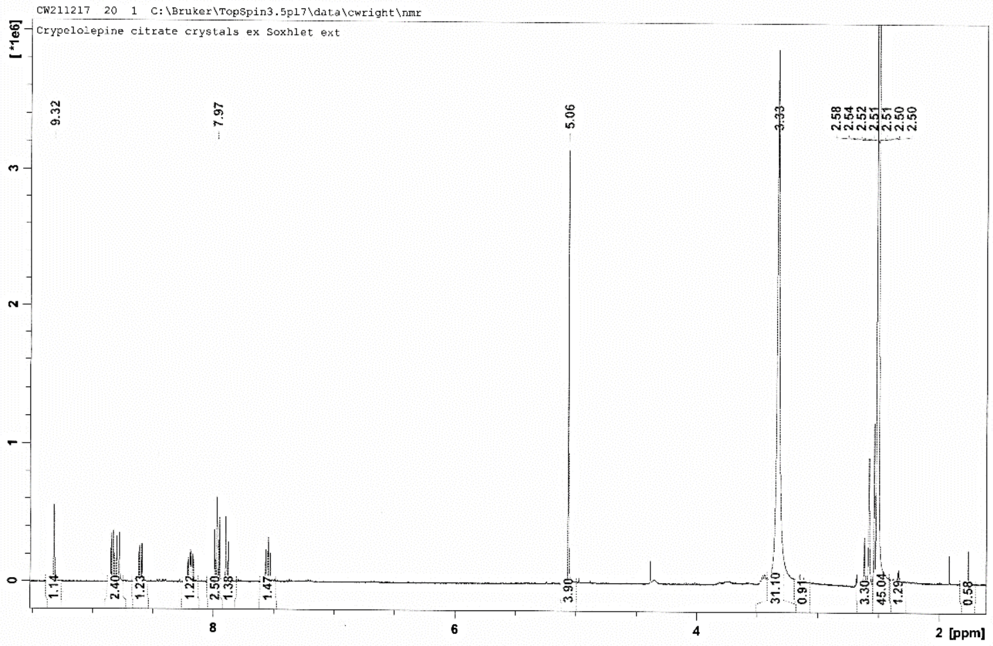

Figure 1B. Expansion of the Aromatic Region of Fig. 1A Showing NMR Signals of 1.

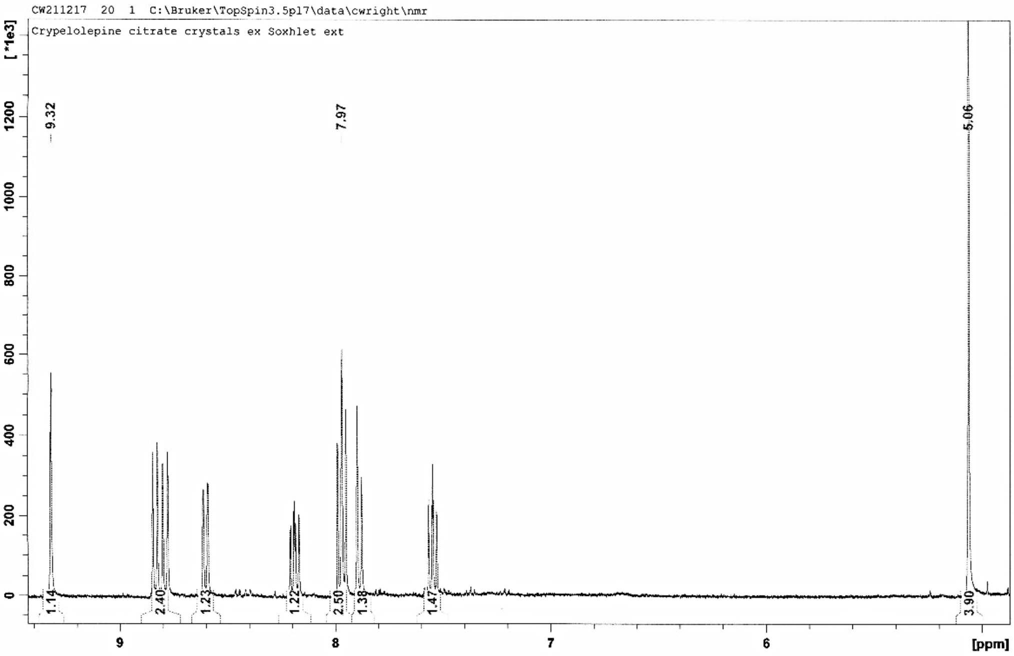

Figure 2. ^{1}H-NMR Spectrum of 7, 9-Dibromocryptolepine (4), in Deuterated TFA.

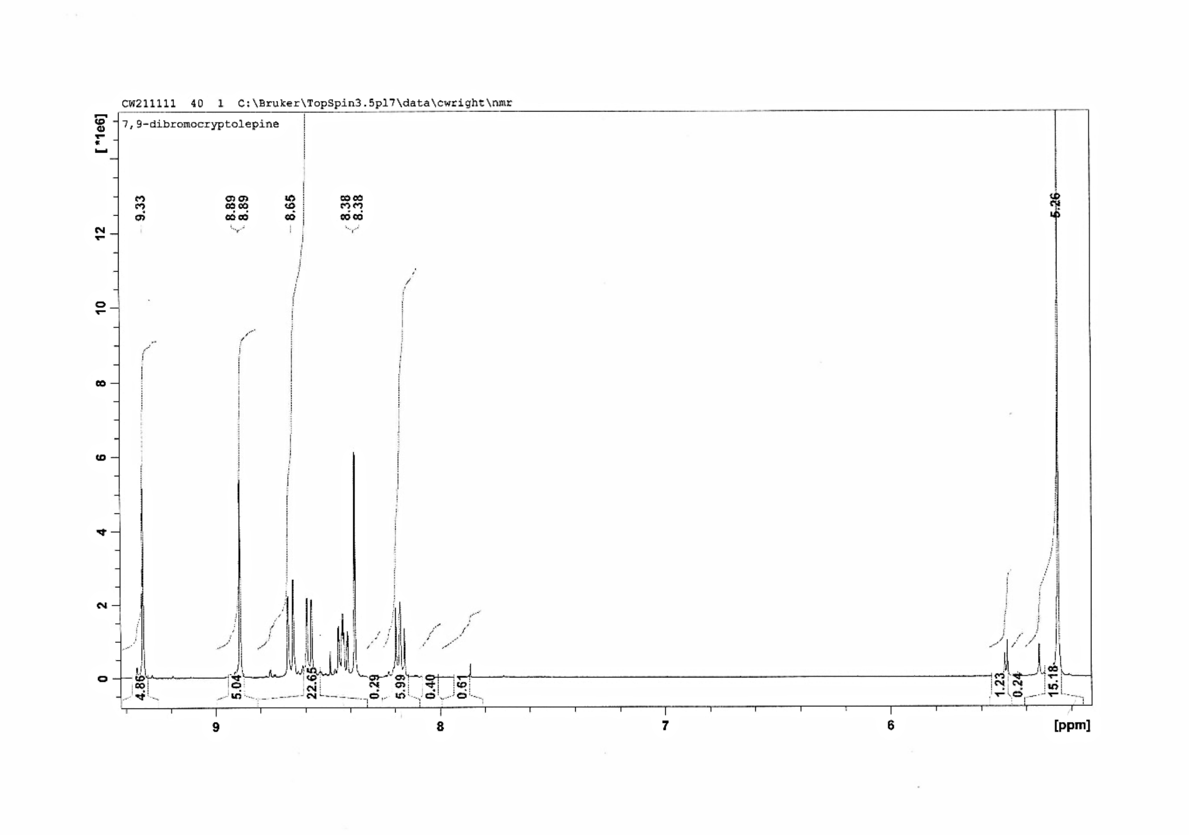

Figure 3. 400 MHz NMR of 7-Iodocryptolepine (5) as Hydrochloride in Deuterated Methanol.

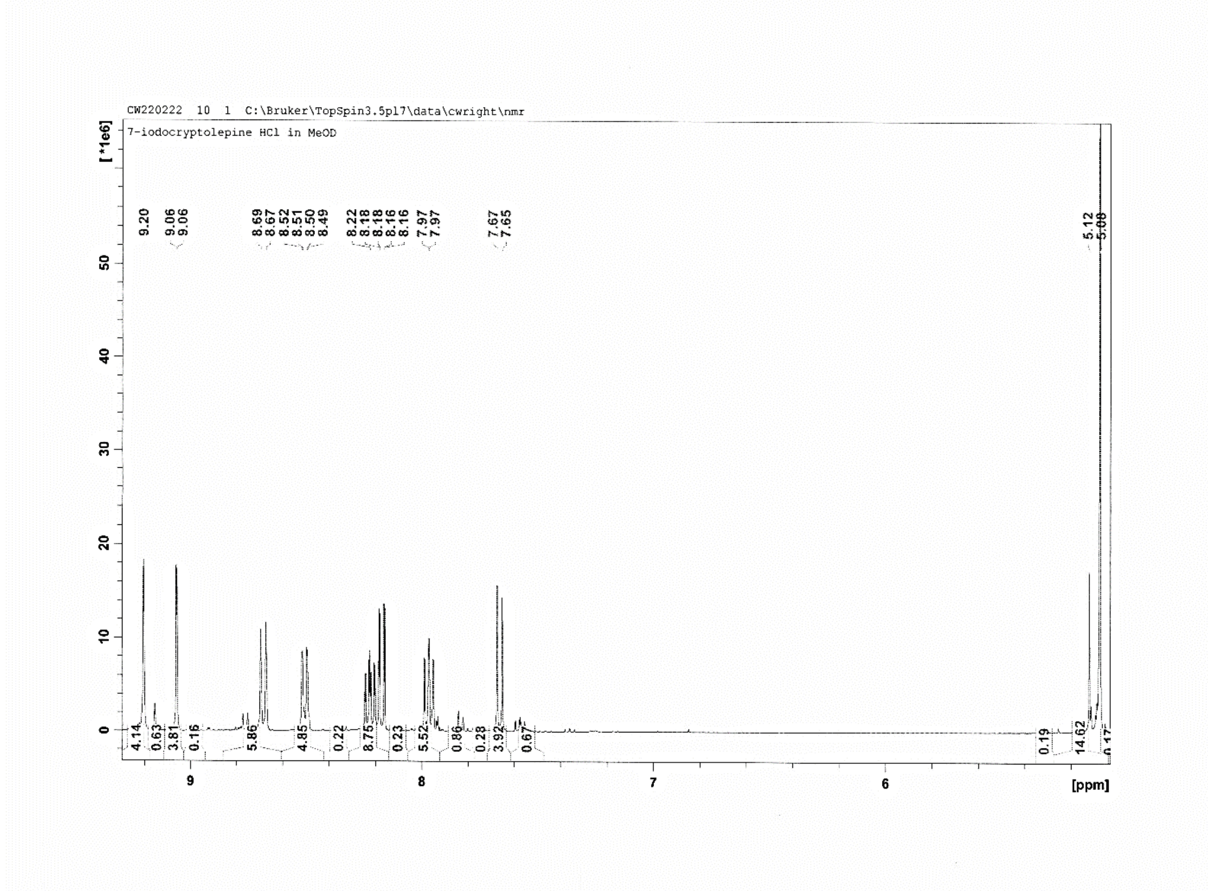

Figure 4. 400 MHz NMR of 7, 9-Iodocryptolepine (6) in Deuterated Methanol.

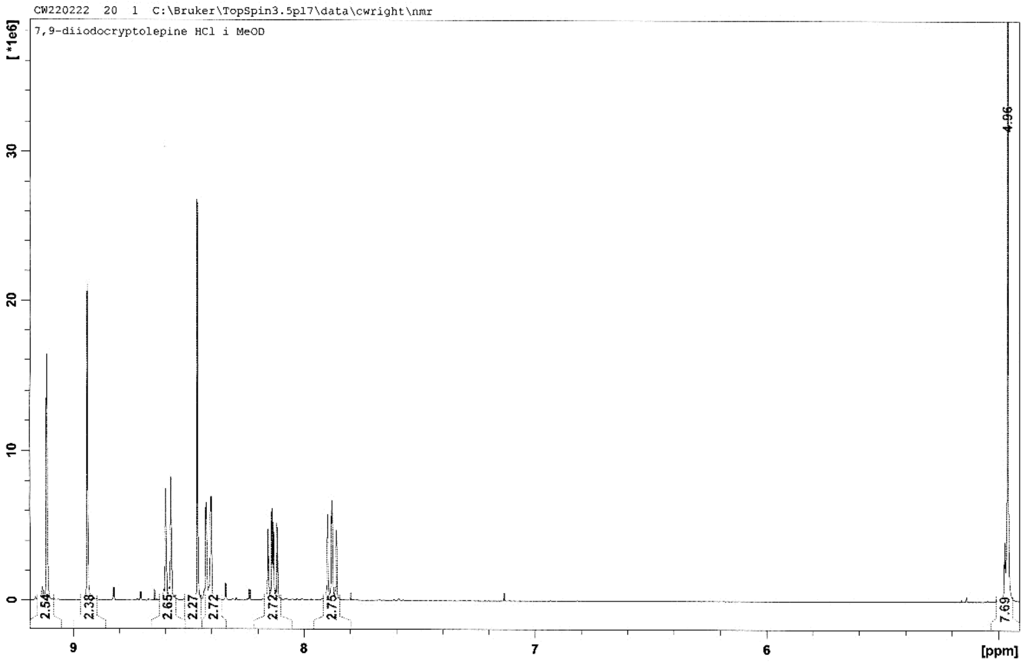

TABLES 1-3 400 MHz NMR data of 4-6, in Deuterated TFA

TABLE 1. 7,9-dibromocryptolepine (**4**)

Position	$\delta_{C;}$	δ_H mult.	COSY	NOESY	HMBC, $^3J_{H\rightarrow C}$
1	115.8	8.63, d, CH	H-2	H-11	C-3, C-11
2	136.9	8.46, t, CH	H-1, H-3		C-11a
3	128.0	8.25, t, CH	H-2, H-4		C-4a
4	130.7	8.65, d, CH	H-3	N-5 CH_3	
4a	126.8	-			
5 (N-CH_3)	39.2	5.32, s, CH_3		H-4, H-6	
5a	136.9	-			
5b	115.0	-			
6	132.1	9.14, s, CH		N-5 CH_3	C5b (^2J), C-7 (^2J), C-8, C-9a
7	137.0	-			
8	150.3	8.77, s, CH			C-5b (^4J), C-6, C-9a
9	134.2	-			
9a	145.8	-			
10 (N)	-	-			
10a	130.1	-			
11	127.7	9.30, CH		H-1	C-5a, 11-a (^2J)
11a	130.1	-			

TABLE 2. 7-iodocryptolepine (**5**)

Position	$\delta_{C;}$	δ_H mult.	COSY	NOESY	HMBC $^3J_{H\rightarrow C}$
1	116.1	8.78, d, CH	H-2	H-11	C-3, C-11
2	132.3	8.60, d, CH	H-1, H-3		C-11a
3	130.3	8.38, t, CH	H-2, H-4		C-1, C-4a
4	134.3	8.82, d, CH	H-3	N-5 CH_3	C-2
4a	136.2	-			
5 (N-CH_3)	38.5	5.45, s, CH_3		H-4, H-6	
5a	136.9	-			
5b	115.8	-			
6	134.0	9.31, s, CH		N-5 CH_3	C-5a, C-5b (^2J), C-9a
7	136.4	-			
8	130.0	8.53, d, CH	H-9		C-6, C-7 (^2J) , C-9a
9	122.0	7.89, d, CH	H-8		C-5b, C-7
9a	145.1	-			
10 (N)	-	-			
10a	133.0				
11	128.2	9.39, s, CH		H-1	C-4a, C-5a
11a	127.1	-			

TABLE 3. 7,9-diiodocryptolepine (**6**)

Position	$\delta_{C;}$	δ_H mult.	COSY	NOESY	HMBC $^3J_{H\rightarrow C}$
1	116.4	8.68, d, CH	H-2	H-11	C-3, C-11
2	134.8	8.45, t, CH	H-1, H-3		C-11a
3	128.3	8.19, t, CH	H-2, H-4		C-1, C-4a
4	130.3	8.65, d, CH	H-3	N-5 CH_3	C-11
4a	137.3	-			
5 (N-CH_3)	38.4	5.26 s, CH_3		H-4, H-6	C-4a, C-5a
5a	132.4	-			
5b	115.4	-			
6	126.1	8.89		N-5 CH_3	C-5a, C-7 (^2J), C-8, C-9a
7	137.1	-			
8	150.1	8.38			C-5b (^4J), C-6, C-9a
9	130.0	-			
9a	142.7	-			
10 (N)	-	-			
10a	134	-			
11	127.5	9.33		H-1	C-4a, C-5a
11a	127	-			

TABLE 4. HRMS Data for 4-6

Compound	Molecular Formula $[M + H]^+$	Calculated	Measured	Δ, ppm
**4**	$C_{16}H_{11}N_2Br_2$	390.92890	390.93055	2.4
**5**	$C_{16}H_{12}N_2I$	359.00456	359.00769	8.7
**6**	$C_{16}H_{11}N_2I_2$	484.90117	484.90768	13.4

Figure 5. Crystallographic Supporting Information

Single-Crystal X-ray Diffraction Measurements and Analysis

X-ray diffraction data on a single crystal (shown below) of diiodocryptolepine hydrochloride monohydrate (**6**) were obtained using an Agilent Oxford Diffraction SuperNova equipped with microfocus Cu and Mo Kα X-ray sources and an Atlas CCD detector. Full spheres of data were collected to 0.84 Å resolution with Cu X-ray radiation and to 0.70 Å resolution with Mo X-ray radiation with each 1° scan frame in ω collected twice. Data collection and reduction was performed using the CrysAlis^{Pro} software package from Oxford Diffraction, versions 1.171.39.46 (Rigaku)[1]. The crystal structure was solved within the Olex2 program suite[2] using the structure solution program ShelXT[3] and refined by least-squares using the refinement program ShelXL.[4] The positions of all atoms except for H were refined freely; although the positions of all H atoms could be seen clearly in the difference map, the positions are fixed at idealized positions. The results from the Cu and Mo refinements were broadly similar. Crystal and molecular structures are illustrated with the program Mercury from CCDC with thermal ellipsoids (including H atoms) shown at 50% probability.[5] The tables below refer to refinements performed using the Mo X-ray data. The corresponding CIF file with *hkl* and intensity data have been deposited at the Cambridge Crystallographic Data Centre with deposition codes 2155899 (Cu) and 2155900 (Mo).

[1] CrysAlis^{Pro} from Rigaku. https://www.rigaku.com/en/products/smc/crysalis.

[2] Dolomanov, O. V.; Bourhis, L. J.; Gildea, R. J., Howard, J. A. K.; Puschmann, H. OLEX2: a Complete Structure Solution, Refinement and Analysis Program. *J. Appl. Crystallogr.*, **2009**, *42* (2), 339–341.

[3] Sheldrick, G. M. *Acta Crystallogr., Sect. A: Found. Adv.*, **2015**, *71* (1), 3–8.

[4] Sheldrick, G. M. Crystal Structure Refinement with SHELXL. *Acta Crystallogr., Sect. C: Struct. Chem.*, **2015**, *71* (1), 3–8.

[5] Macrae, C. F.; Bruno, I. J.; Chisholm, J. A.; Edgington, P. R.; McCabe, P.; Pidcock, E.; Rodriguez-Monge, L.; Taylor, R.; van de Streek, J.; Wood, P. A. Mercury CSD 2.0 – New Features for the Visualization and Investigation of Crystal Structures. *J. Appl. Crystallogr.*, **2008**, *41* (2), 466–470.

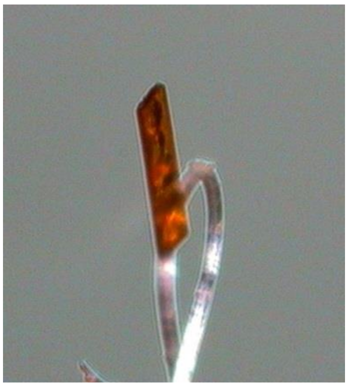

View of the 0.4 by 0.09 by 0.02 mm sized crystal used in the diffraction experiment.

Table S5a. Crystal data and structure refinement for 7,9-diiodocryptolepine hydrochloride monohydrate (**6**) at 150 K.

Identification code	exp_1370
Empirical formula	$C_{16}H_{13}ClI_2N_2O$
Formula weight	538.53
Temperature / K	150
Crystal system	monoclinic
Space group	$P2_1/c$
a / Å	9.3791(3)
b / Å	25.3783(6)
c / Å	7.4188(2)
α / °	90
β / °	105.778(3)
γ / °	90
Volume / Å^3	1699.33(9)
Z	4
ρ_{calc} / g cm^{-3}	2.105
μ / mm^{-1}	3.861
F(000)	1016.0
Crystal size / mm^3	0.401 × 0.091 × 0.02
Radiation	Mo Kα (λ = 0.71073 Å)
2θ range for data collection / °	6.444 to 58.256
Index ranges	$-12 \le h \le 12$, $-34 \le k \le 34$, $-10 \le l \le 10$
Reflections collected	74763
Independent reflections	4554 [R_{int} = 0.0390, R_{sigma} = 0.0142]
Data/restraints/parameters	4554/0/203
Goodness-of-fit on F^2	1.091
Final R indexes [$I \ge 2\sigma(I)$]	R_1 = 0.0268, wR_2 = 0.0694
Final R indexes [all data]	R_1 = 0.0309, wR_2 = 0.0715
Largest diff. peak/hole / e Å^{-3}	1.13/−0.89

Table S5b. Fractional atomic coordinates and equivalent isotropic displacement parameters for 7,9-diiodocryptolepine hydrochloride monohydrate (**6**) at 150 K. U_{eq} is defined as ⅓ of the trace of the orthogonalised U_{ij} tensor.

Atom	*x*	*y*	*z*	*U*(eq) / Å^2
I(1)	0.70776(2)	0.80635(2)	0.88772(3)	0.0255(6)
I(2)	0.15583(3)	0.69899(2)	0.43173(4)	0.0404(8)
N(1)	0.3808(3)	0.5962(9)	0.6139(3)	0.0209(4)
N(2)	0.7187(2)	0.5601(9)	0.9368(3)	0.0176(4)
C(1)	0.4362(3)	0.64534(11)	0.6643(4)	0.0199(5)
C(2)	0.3712(3)	0.69441(11)	0.6071(4)	0.0236(5)
C(3)	0.4489(3)	0.73957(12)	0.6737(4)	0.0244(5)
C(4)	0.5917(3)	0.73655(11)	0.7979(4)	0.0214(5)
C(5)	0.6565(3)	0.68881(11)	0.8616(4)	0.0210(5)
C(6)	0.5784(3)	0.64243(11)	0.7946(4)	0.0188(5)
C(7)	0.6053(3)	0.58693(10)	0.8258(3)	0.0173(5)
C(8)	0.4800(3)	0.55968(10)	0.7118(4)	0.0182(5)
C(9)	0.4720(3)	0.50586(11)	0.7108(4)	0.0209(5)
C(10)	0.5899(3)	0.47825(11)	0.8312(4)	0.0193(5)
C(11)	0.5863(3)	0.42226(11)	0.8420(4)	0.0245(5)
C(12)	0.6976(4)	0.39536(11)	0.9651(4)	0.0269(6)
C(13)	0.8174(3)	0.42242(12)	1.0842(4)	0.0248(6)
C(14)	0.8255(3)	0.47679(11)	1.0766(4)	0.0220(5)
C(15)	0.7137(3)	0.50546(10)	0.9486(4)	0.0189(5)
C(16)	0.8503(3)	0.59006(11)	1.0403(4)	0.0227(5)
Cl(1)	−0.16212(7)	0.57480(3)	0.53810(9)	0.02287(13)
O(1)	0.1230(2)	0.5489(9)	0.4056(3)	0.0262(4)
H(1)	0.2975	0.5893	0.5341	0.025
H(3)	0.4067	0.7723	0.6363	0.029
H(5)	0.7497	0.6874	0.9469	0.025
H(9)	0.3916	0.4882	0.6334	0.025
H(11)	0.5075	0.4038	0.7645	0.029
H(12)	0.6939	0.3588	0.9700	0.032
H(13)	0.8916	0.4037	1.1685	0.030
H(14)	0.9049	0.4945	1.1561	0.026
H(16A)	0.8761	0.6153	0.9580	0.034
H(16B)	0.9317	0.5663	1.0865	0.034
H(16C)	0.8288	0.6081	1.1436	0.034
H(1A)	0.0515	0.5563	0.4513	0.039
H(1B)	0.1322	0.5156	0.4167	0.039

Table S5c. Anisotropic displacement parameters for 7,9-diiodocryptolepine hydrochloride monohydrate (**6**) at 150 K. The anisotropic displacement factor exponent has the form: $-2\pi^2[h^2a^{*2}U_{11}+2hka^*b^*U_{12}+\ldots]$.

Atom	U_{11} / Å^2	U_{22} / Å^2	U_{33} / Å^2	U_{23} / Å^2	U_{13} / Å^2	U_{12} / Å^2
I(1)	0.0298(11)	0.0229(10)	0.0253(10)	0.0007(7)	0.0101(8)	−0.0022(7)
I(2)	0.0269(12)	0.0368(13)	0.0471(14)	0.0022(9)	−0.0075(10)	0.0060(8)
N(1)	0.0160(10)	0.0246(12)	0.0194(10)	0.0007(9)	0.0003(8)	−0.0015(8)
N(2)	0.0139(10)	0.0215(11)	0.0173(10)	−0.0036(8)	0.0040(8)	−0.0005(8)
C(1)	0.0183(12)	0.0234(13)	0.0181(11)	0.0007(9)	0.0052(9)	0.0002(10)
C(2)	0.0182(12)	0.0282(14)	0.0230(13)	0.0047(10)	0.0033(10)	0.0044(10)
C(3)	0.0241(13)	0.0235(13)	0.0255(14)	0.0030(10)	0.0068(11)	0.0039(10)
C(4)	0.0240(13)	0.0204(12)	0.0212(12)	−0.0003(10)	0.0087(10)	−0.0010(10)
C(5)	0.0209(13)	0.0234(13)	0.0192(12)	0.0005(10)	0.0064(10)	−0.0001(10)
C(6)	0.0177(12)	0.0216(12)	0.0174(11)	0.0025(9)	0.0055(9)	0.0016(9)
C(7)	0.0152(11)	0.0220(12)	0.0153(11)	−0.0011(9)	0.0049(9)	−0.0010(9)
C(8)	0.0159(11)	0.0240(13)	0.0154(11)	−0.0008(9)	0.0052(9)	−0.0005(9)
C(9)	0.0188(12)	0.0259(13)	0.0181(12)	−0.0034(10)	0.0049(10)	−0.0048(10)
C(10)	0.0209(12)	0.0212(12)	0.0174(11)	−0.0010(9)	0.0080(10)	−0.0016(10)
C(11)	0.0272(14)	0.0212(13)	0.0261(13)	−0.0033(10)	0.0088(11)	−0.0040(11)
C(12)	0.0355(16)	0.0182(13)	0.0297(15)	0.0002(11)	0.0134(12)	0.0016(11)
C(13)	0.0249(13)	0.0256(14)	0.0245(13)	0.0061(11)	0.0078(11)	0.0025(11)
C(14)	0.0195(12)	0.0246(13)	0.0223(12)	−0.0008(10)	0.0062(10)	0.0017(10)
C(15)	0.0196(12)	0.0202(12)	0.0176(11)	−0.0011(9)	0.0063(9)	0.0011(9)
C(16)	0.0158(12)	0.0239(13)	0.0252(13)	−0.0036(10)	0.0003(10)	−0.0022(10)
Cl(1)	0.0206(3)	0.0217(3)	0.0257(3)	−0.0007(2)	0.0052(2)	0.0027(2)
O(1)	0.0202(10)	0.0266(10)	0.0295(10)	0.0005(8)	0.0027(8)	0.0003(8)

Table S5d. Selected bond lengths for 7,9-diiodocryptolepine (**6**) at 150 K.

Atom — Atom	Length / Å	Atom — Atom	Length / Å
I(1) — C(4)	2.091(3)	C(5) — C(6)	1.404(4)
I(2) — C(2)	2.087(3)	C(6) — C(7)	1.439(4)
N(1) — C(1)	1.364(4)	C(7) — C(8)	1.427(4)
N(1) — C(8)	1.373(3)	C(8) — C(9)	1.368(4)
N(2) — C(7)	1.341(3)	C(9) — C(10)	1.405(4)
N(2) — C(15)	1.390(3)	C(10) — C(11)	1.424(4)
N(2) — C(16)	1.475(3)	C(10) — C(15)	1.426(4)
C(1) — C(2)	1.401(4)	C(11) — C(12)	1.368(4)
C(1) — C(6)	1.420(4)	C(12) — C(13)	1.405(4)
C(2) — C(3)	1.376(4)	C(13) — C(14)	1.384(4)
C(3) — C(4)	1.406(4)	C(14) — C(15)	1.410(4)
C(4) — C(5)	1.380(4)		

Table S5e. Selected bond angles for 7,9-diiodocryptolepine hydrochloride monohydrate (**6**) at 150 K.

Atom — Atom — Atom	Angle / °	Atom — Atom — Atom	Angle / °
C(1) — N(1) — C(8)	108.6(2)	N(2) — C(7) — C(6)	132.2(2)
C(7) — N(2) — C(15)	120.7(2)	N(2) — C(7) — C(8)	120.4(2)
C(7) — N(2) — C(16)	118.0(2)	C(8) — C(7) — C(6)	107.4(2)
C(15) — N(2) — C(16)	121.3(2)	N(1) — C(8) — C(7)	108.5(2)
N(1) — C(1) — C(2)	128.9(3)	N(1) — C(8) — C(9)	130.0(2)
N(1) — C(1) — C(6)	110.9(2)	C(9) — C(8) — C(7)	121.5(2)
C(2) — C(1) — C(6)	120.2(3)	C(8) — C(9) — C(10)	117.6(2)
C(1) — C(2) — I(2)	120.4(2)	C(9) — C(10) — C(11)	120.5(2)
C(3) — C(2) — I(2)	120.4(2)	C(9) — C(10) — C(15)	121.1(2)
C(3) — C(2) — C(1)	119.1(3)	C(11) — C(10) — C(15)	118.4(2)
C(2) — C(3) — C(4)	120.5(3)	C(12) — C(11) — C(10)	120.7(3)
C(3) — C(4) — I(1)	118.9(2)	C(11) — C(12) — C(13)	120.7(3)
C(5) — C(4) — I(1)	119.5(2)	C(14) — C(13) — C(12)	120.3(3)
C(5) — C(4) — C(3)	121.6(3)	C(13) — C(14) — C(15)	120.2(3)
C(4) — C(5) — C(6)	118.4(3)	N(2) — C(15) — C(10)	118.7(2)
C(1) — C(6) — C(7)	104.6(2)	N(2) — C(15) — C(14)	121.7(2)
C(5) — C(6) — C(1)	120.0(2)	C(14) — C(15) — C(10)	119.6(2)
C(5) — C(6) — C(7)	135.4(2)		

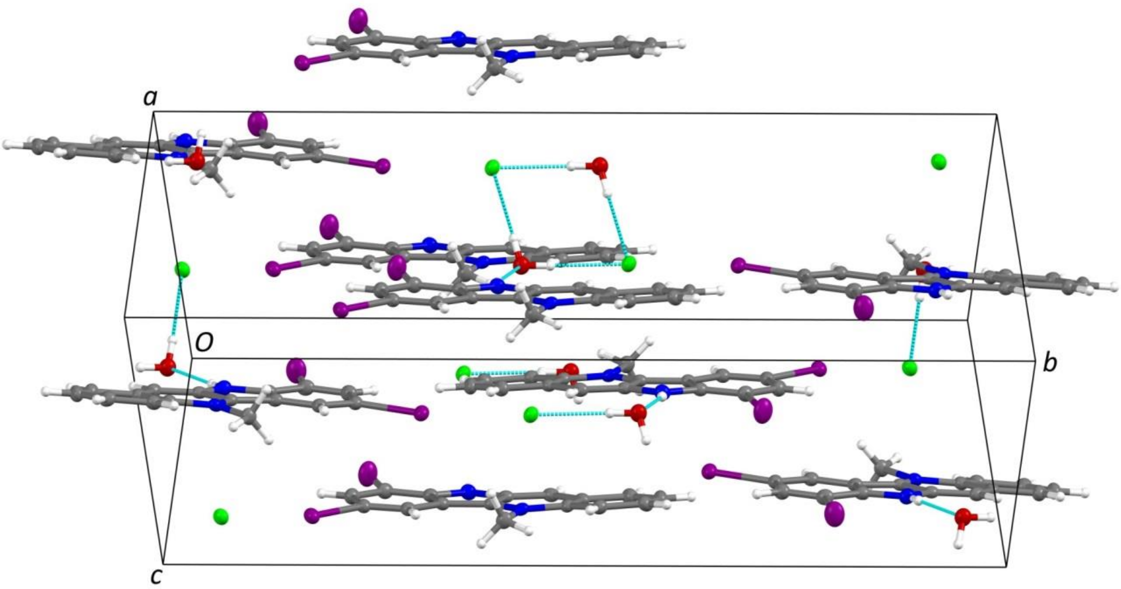

Figure 5a. The crystal structure of 7,9-diiodocryptolepine hydrochloride monohydrate (**6**) at 150 K. The molecules lie in sheets connected via a hydrogen bonded network formed by the water molecule, protonated N atom, and the chloride anion.

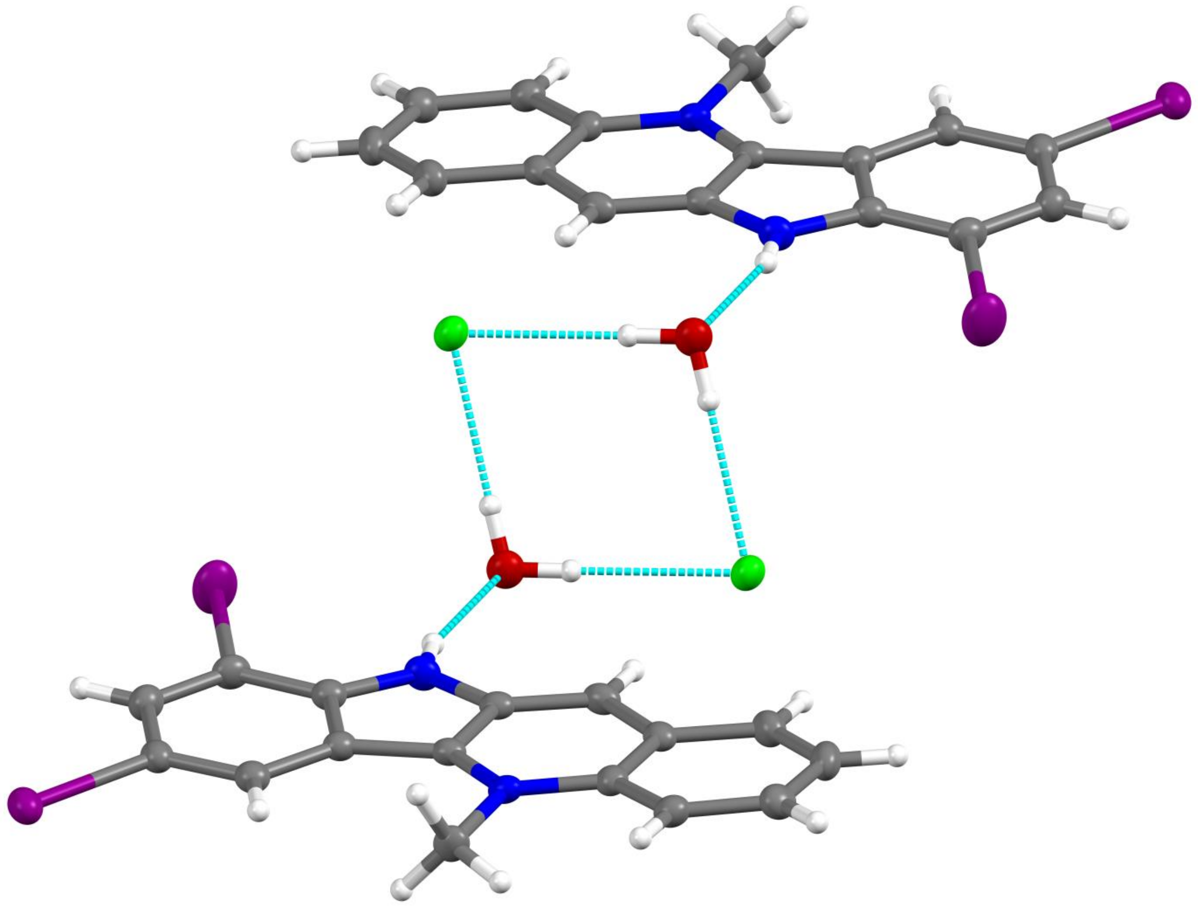

Figure 5b. A detailed view of the hydrogen bonding network formed by the water molecule, protonated N atom, and the chloride anion. Although the positions of the H atoms were not refined, Fourier difference maps showed their positions clearly.

Figure 6. Summary of the Dose-Response Curves Obtained for 1, 2 and 6 Against Late Stage Gametocytes

Table 1: Summary of the dose response curves obtained on late stage gametocytes. Compounds were screened for IC_{50} against late gametocytes (>90% stage IV/V gametocytes) using the luciferase assay platform.

<table>
<tr><th>Compound</th><th colspan="2">Late stage gametocytes (>90% stage IV/V gametocytes)</th><th>Late stage gametocyte comments</th></tr>
<tr><td rowspan="10">Cryptolepine</td><td colspan="2">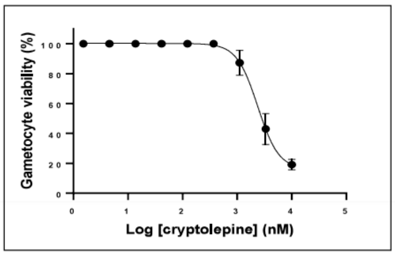</td><td>IC_{50} = 2.4 µM, No bottom plateau, inhibition max @ 10 µM = 80.86%</td></tr>
<tr><td>IC_{50} (nM, ± SEM)</td><td>2401 ± 304</td><td rowspan="9">To obtain full dose response curve, a higher starting concnetraion is required.</td></tr>
<tr><td>Range</td><td>10 µM - 1.5 nM</td></tr>
<tr><td>IC_{50} n =</td><td>3</td></tr>
<tr><td>IC_{50} R^2</td><td>0.9830</td></tr>
<tr><td>IC_{50} z-factor</td><td>0.88</td></tr>
<tr><td>Max inhibition (%)</td><td>80.86</td></tr>
<tr><td>Hill slope</td><td>-2,256</td></tr>
<tr><td>MB (%@5 µM)</td><td>95.9</td></tr>
<tr><td>MMV048 (%@5 µM)</td><td>97.5</td></tr>
<tr><td rowspan="10">2,7-dibromocryptolepine</td><td colspan="2">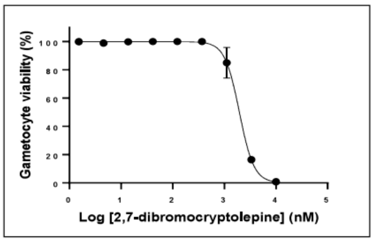</td><td>IC_{50} = 2 µM, inhibition max @ 10 µM = 99.23%</td></tr>
<tr><td>IC_{50} (nM, ± SEM)</td><td>1990 ± 169</td><td rowspan="9"></td></tr>
<tr><td>Range</td><td>10 µM - 1.5 nM</td></tr>
<tr><td>IC_{50} n =</td><td>3</td></tr>
<tr><td>IC_{50} R^2</td><td>0.9935</td></tr>
<tr><td>IC_{50} z-factor</td><td>0.88</td></tr>
<tr><td>Max inhibition (%)</td><td>99.23</td></tr>
<tr><td>Hill slope</td><td>-3,078</td></tr>
<tr><td>MB (%@5 µM)</td><td>95.9</td></tr>
<tr><td>MMV048 (%@5 µM)</td><td>97.5</td></tr>
<tr><td rowspan="10">7,9-diiodocryptolepine</td><td colspan="2">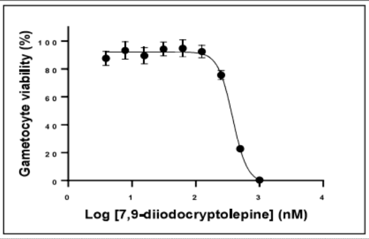</td><td>IC_{50} = 2.2 µM, inhibition max @ 10 µM = 99.60%</td></tr>
<tr><td>IC_{50} (nM, ± SEM)</td><td>2152 ± 23.5</td><td rowspan="9"></td></tr>
<tr><td>Range</td><td>10 µM - 1.5 nM</td></tr>
<tr><td>IC_{50} n =</td><td>3</td></tr>
<tr><td>IC_{50} R^2</td><td>0.9828</td></tr>
<tr><td>IC_{50} z-factor</td><td>0.9</td></tr>
<tr><td>Max inhibition (%)</td><td>99.60</td></tr>
<tr><td>Hill slope</td><td>-3,779</td></tr>
<tr><td>MB (%@5 µM)</td><td>95.3</td></tr>
<tr><td>MMV048 (%@5 µM)</td><td>96.2</td></tr>
</table>

Figure 7. Summary of the Dose Response Curves Obtained from 2, 5, 6 and Atovaquone as Positive Control with Liver stage *P. beghei* and HepG2 cells .

Synonyms	Liver Stage P. berghei Luciferase: IC50 (uM)	Liver Stage P. berghei Luciferase: Dose-response Plot	Liver Stage HepG2 Toxicity Bioluminescence Assay: IC50 (uM)	Liver Stage HepG2 Toxicity Bioluminescence Assay: Dose-response Plot
7-iodo crytptolepine citrate	14.9	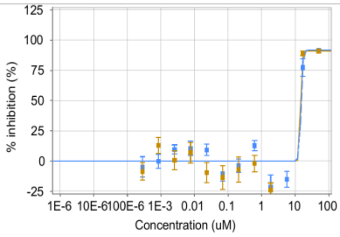	*6.35*	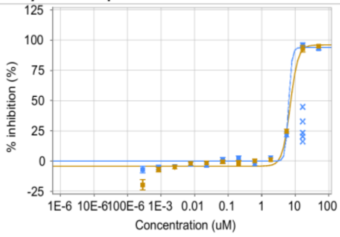
7,9 dibromo cryptolepine citrate	6	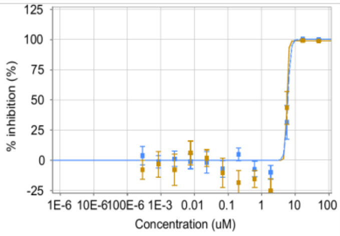	7.44	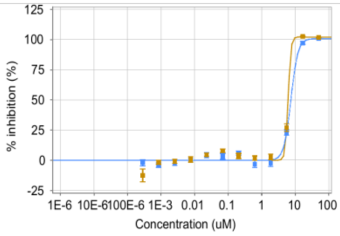
2,7-dibromoCryptolepine hydrochloride (analog)	*2.76*	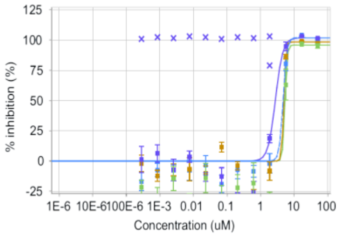	3.66	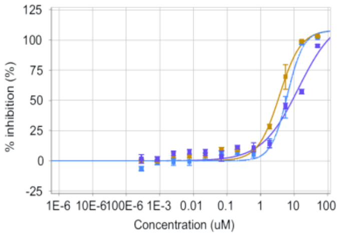
Atovaquone, CBR-001-040-600-5, MMV000046	0.00048	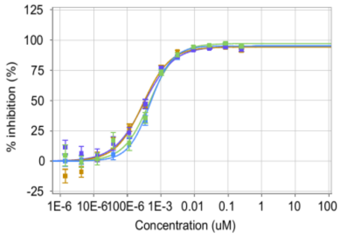	> 0.250	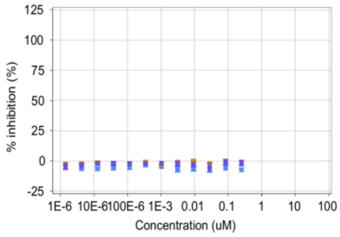

Figure 8. Oral Toxicity Data for 2 and 4-6 at 20 mg, 50 mg and 100 mg/kg Bodyweight Daily for 3 days in Mice.

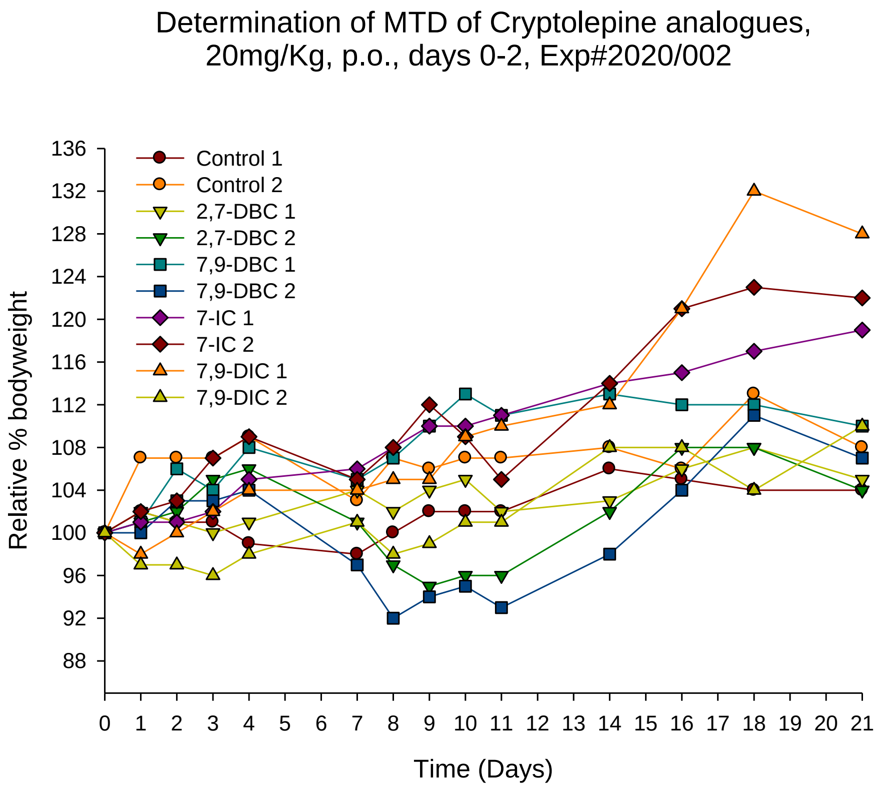

Determination of MTD of Cryptolepine analogues, 50mg/Kg, p.o., days 0-2, Exp#2020/004

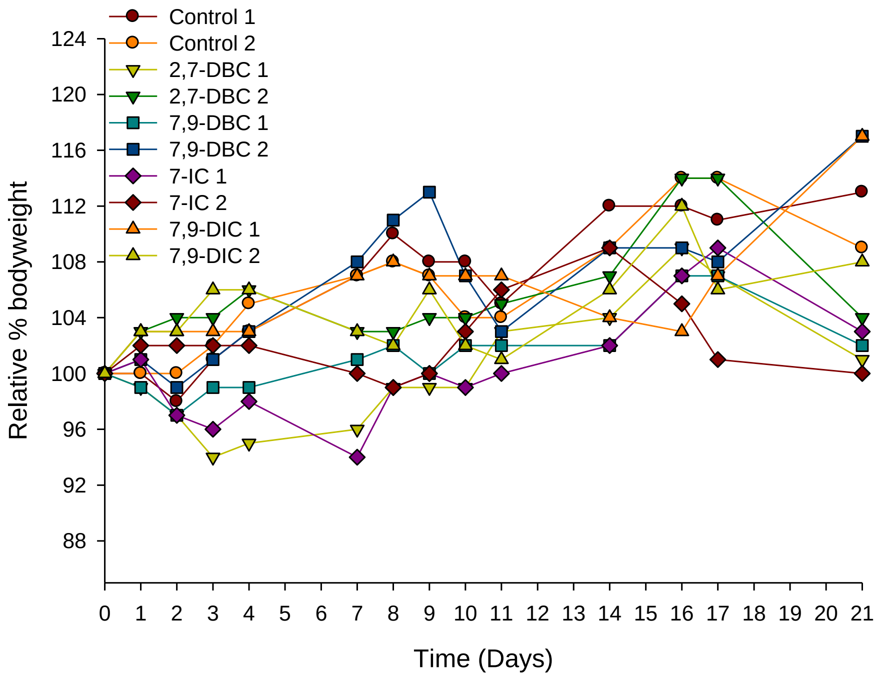

Determination of MTD of Cryptolepine analogues, 100mg/Kg, p.o., days 0-2, Exp#2020/006

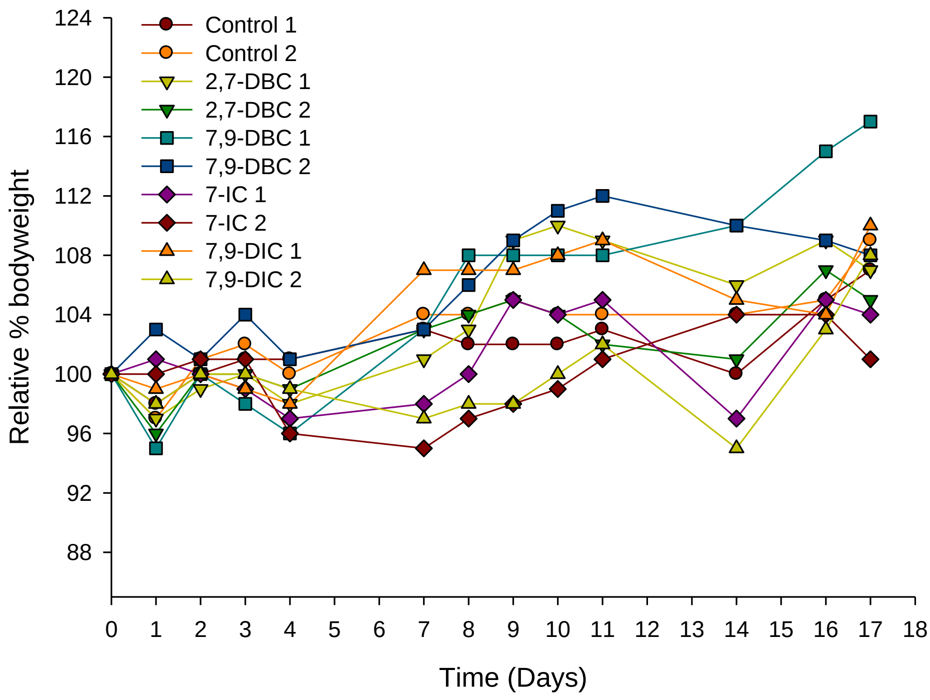

Supplement: Supplementary file 1 [file DataSheet1.pdf]
